# CQMUH-011 mitigated LPS-induced acute lung injury in neonatal rabbits

**DOI:** 10.1038/s41598-026-49167-x

**Published:** 2026-04-19

**Authors:** Yaling Xu, Xiaojing Guo, Xiao Han, Xiangnan Hu, Bin Gao, Bo Sun, Huayan Zhang

**Affiliations:** 1 Division of Neonatology and Center for Newborn Care, Guangzhou Women and Children’s Medical Center, Guangzhou Medical University, Guangdong, 510623 China; 2 The Laboratory of Neonatal Diseases of National Commission of Health, National Children’s Medical Center, Children’s Hospital of Fudan University, Shanghai, China; 3 Institute of Pediatric, Guangzhou Women and Children’s Medical Center, Guangzhou Medical University, Guangzhou, Guangdong China; 4 Department of Pharmacology, the Key Laboratory of Biochemistry and Molecular Pharmacology, Chongqing Medical University, Chongqing, China; 5 Food and Drug College of Yunnan, Yuxi Agriculture Vocation-Technical College, Yunnan, China; 6 Division of Neonatology, Department of Pediatrics, Children’s Hospital of Philadelphia, University of Pennsylvania Perelman School of Medicine, Philadelphia, PA USA

**Keywords:** Neonatal sepsis, Acute lung injury, Anti-inflammation, Lung protection, CQMUH-011, Cell biology, Diseases, Drug discovery, Immunology, Medical research

## Abstract

Neonatal sepsis-associated acute lung injury (ALI) remains a major clinical challenge. This study investigated the protective effects of a novel small-molecule compound, CQMUH-011, in a neonatal rabbit model of hyper-acute endotoxin shock with secondary ALI. Neonatal rabbits were intraperitoneally injected with lipopolysaccharide (50 mg/kg), followed by CQMUH-011 (75, 225, 675 µg/kg) or dexamethasone (Dex, 1.5 mg/kg) at 0.5 h. Over a 10-hour observation period, despite a non-significant trend in overall survival, CQMUH-011 dose-dependently mitigated neurological deficits, restored blood gas balance, enriched the phospholipid pool, reduced inflammatory lung injury and improved alveolar expansion. At the mRNA level, CQMUH-011 suppressed proinflammatory cytokines and upregulated surfactant-associated enzymes. The lung-protective effects of CQMUH-011 were comparable to **t**hose of Dex, or better on selected endpoints. Exploratory transcriptomic profiling suggested CQMUH-011 may function by downregulating multiple inflammatory pathways concurrent with the potential modulation of phosphatidylinositol 3-kinase/protein kinase B-dependent cytoprotection, cytoskeletal remodeling, and intercellular junction reinforcement. However, these transcriptomic signatures currently lack confirmatory protein-level testing. In conclusion, CQMUH-011 demonstrates preliminary lung-protective effects in this acute neonatal endotoxin shock paradigm. Rather than a definitive therapeutic candidate, CQMUH-011 represents an early-stage proof-of-concept molecule requiring extensive future pharmacokinetic characterization and mechanistic validation to establish its translational potential.

**Supplementary Information:**

The online version contains supplementary material available at 10.1038/s41598-026-49167-x.

## Introduction

Neonatal infections remain a major contributor to morbidity and mortality worldwide, particularly in preterm infants^1–3^. The underdeveloped immune systems and impaired infection defenses of newborns heighten their vulnerability to uncontrolled inflammation, which often rapidly escalates into life-threatening conditions like neonatal sepsis^2^. While sepsis-induced acute respiratory distress syndrome (ARDS) has been extensively characterized in adults and older pediatric populations^4,5^, the distinct clinical entity of neonatal ARDS has been formally established by the Montreux definition and recognized for its significant disease burden^5,6^. To investigate the underlying pathogenesis of this condition, preclinical studies utilize models of experimental acute lung injury (ALI), which closely recapitulate the pathophysiological damage patterns seen during sepsis, including inflammatory cell infiltration, leaky blood vessels, and the breakdown of lung epithelial-endothelial barriers^4,6^. In addition, critical lung developmental processes, such as alveologenesis, angiogenesis, and extracellular matrix deposition, can be disrupted during neonatal sepsis, thereby culminating in chronic lung disease with lifelong pulmonary sequelae^7^.

Currently, the management of neonatal sepsis-induced ALI or ARDS mainly relies on antimicrobial interventions and mechanical ventilation^5,6^. With limited choices of targeted anti-inflammatory therapies, glucocorticoids remain as the main pharmacological agents used both in adult and pediatric populations^4,5^. However, the use of glucocorticoids in the neonatal population, especially premature infants, is limited due to both short-term complications (e.g., hyperglycemia, gastrointestinal bleeding) and the potential for adverse long-term developmental consequences like neurodevelopmental delays and metabolic syndrome^8^. Finding effective and safer anti-inflammatory strategies is therefore crucial for the neonatal population.

CQMUH-011 is a novel adamantane sulfonamide derivative (molecular formula C23H30N3O4S, molecular weight 457 g/mol)^9^ that has demonstrated notable immunomodulatory capabilities. Preclinical studies have demonstrated its capacity to suppress lipopolysaccharide (LPS)-induced inflammatory responses in macrophage and microglial cellular environments. Comparative studies have revealed anti-inflammatory efficacy comparable to that of dexamethasone across experimental models, including ischemic brain injury, fulminant hepatic failure, and inflammatory arthritis^10–12^. However, the therapeutic potential of this compound for treating sepsis-induced neonatal ALI and its mechanistic basis remain poorly characterized. The neonatal rabbit model (postnatal days 5–7) was specifically chosen for this study because its pulmonary developmental stage (active alveolarization) and surfactant biology closely mirror those of human near-term and term infants, making it a highly robust and clinically translatable model for neonatal lung injury^13^. Therefore, this study investigates the therapeutic effects of CQMUH-011 on LPS-induced ALI in neonatal rabbits and explores potential associated molecular mechanisms through transcriptomic profiling.

## Materials and methods

### Reagents and animals

CQMUH-011 (purity > 95%) was provided by the patent owner (Patent Application No. 201610818842.7, P.R.C) and a co-investigator of the study (Xiangnan Hu). Dexamethasone (Dex) (purity ≥ 98%) was purchased from Beijing Solarbio Science & Technology Co., Ltd (Beijing, China). LPS (*Escherichia coli*, O111:B4, L2630-100MG) was obtained from Sigma-Aldrich (St. Louis, USA). Neonatal rabbits at postnatal age of 5–7 days and body weight (BW) of 80–100 g were obtained from Shanghai Songlian Experimental Animal Center. The study protocol was approved by the Ethics Committees of Children’s Hospital of Fudan University (No.2024241) and Guangzhou Women and Children’s Medical Center (No.KTDW-2025-01500). All methods were performed in accordance with the relevant guidelines and regulations.

### Animal experimental protocol

The study was designed and reported in accordance with the ARRIVE guidelines 2.0 (https://arriveguidelines.org/arrive-guidelines). To minimize potential maternal and genetic litter effects, pups of mixed sexes from multiple litters were pooled together. After 12 h of environmental adaptation, neonatal rabbits were randomly assigned to receive saline, LPS, LPS with Dex or LPS with various doses of CQMUH-011 treatment via intraperitoneal injections. Randomization was performed using a computer-generated random number sequence, and allocation concealment was maintained by an independent investigator who prepared the coded interventions. Six treatment groups were established, with 14 to 16 animals per group: (a) vehicle-treated control (Saline); (b) LPS (LPS 50 mg/kg)^13^; (c) LPS + Dex (1.5 mg/kg) (LPS-Dex); and (d-f) LPS + CQMUH-011 (75, 225, and 675 µg/kg, respectively) (LPS-011-75, LPS-011-225, LPS-011-675). The high intraperitoneal LPS dose (50 mg/kg) was chosen to induce severe hyper-acute endotoxin shock and secondary ALI comparable to the established 20 mg/kg intravenous model neonatal rabbit model^13^, targeting a 40–50% mortality rate at 10 h to simulate an unsupported, natural disease course. Animals in groups c-f received either Dex or CQMUH-011 half an hour post-LPS administration. The dexamethasone dose (1.5 mg/kg) was selected based on previous neonatal rabbit studies demonstrating reliable anti-inflammatory efficacy within this dose range, and was administered 0.5 h post-LPS to model early intervention in the acute phase of sepsis.

Animals were continuously monitored for health status and subjected to neurobehavioral assessment for up to 10 h or until death, whichever came first. At study termination, blood samples, bronchoalveolar lavage fluid (BALF) and lung tissues were collected for downstream analyses. Surviving animals were deeply anesthetized with 5% isoflurane in an induction chamber and then euthanized by decapitation. Complete anesthesia was confirmed by the loss of toe-pinch reflex. The right lung was immediately removed and stored at -80℃ for molecular analyses, including reverse transcription polymerase chain reaction (RT-PCR) and transcriptomic profiling. The accessory lobe of the right lung and the left lung were randomly assigned to histopathological or biochemical measurements (*n* = 5–8 per group).

### Health status monitoring

All experimental animals were housed in a temperature-controlled incubator to ensure consistent physiological thermal regulation. Survival status was assessed every 30 min through the evaluation of animals’ voluntary motor activity, peripheral tissue perfusion (via lip, limb, and trunk coloration), and respiration (evaluated via thoracic movement patterns). Survival time was recorded during the 10-hour observation period. At the end of the experiment, surviving animals underwent cardiac function evaluation via electrocardiography (ECG) using a bioelectric amplifier (ADInstruments), and core body temperature measurement through rectal thermometry using a calibrated mercury thermometer.

### Neurobehavioral assessment

Central injury score (CIS) was evaluated using a validated neurobehavioral scale adapted from Derrick et al.^14^ For each animal, the testing was videotaped and scored every half hour until study termination (10 h or death) on a scale of 0–3 (0 being the worst; 3 being the best). All videotaped evaluations were conducted by two independent, trained investigators who were strictly blinded to the experimental conditions and group allocations of the individual animals. To be more specific, a score of 3 represents good response to stimulation, normal crawling, quick to roll over after lying down; 2 represents average response to stimulation, reduced crawling, unable to roll over immediately after lying down; 1 represents poor response to stimulation, significantly reduced crawling, unable to roll over after lying down; and 0 represents no response to stimulation. For animals that died before the 10-hour observation endpoint, a score of 0 was recorded at the time point closest to death, reflecting the most severe neurological impairment. No further scores were assigned after death, and the animal’s data were treated as missing at subsequent time points in the longitudinal analysis.

### Blood gas

The mixed venous blood samples were collected via cardiac puncture from the right ventricle for blood gas analysis on ABL 90 Series Analyzer (Radiometer Medical, Copenhagen, Denmark). The potential of hydrogen (pH), partial oxygen pressure (pO_2_), partial carbon dioxide pressure (pCO_2_), and lactate concentration were recorded.

### Histological analysis

The accessory lobe of the right lung was removed to measure the wet-to-dry weight ratio (W/D) to estimate fluid content in the whole lung tissue. In each group, the left lung was fixed *en bloc* for hematoxylin-eosin staining. For histological quantification, fifty random fields from each animal’s lung were examined at ×200 magnification to conduct a semi-quantitative evaluation of the lung injury score (LIS), alveolar expansion (Vv), and the coefficient of variation of alveolar expansion [CV(Vv)]^15,16^. Importantly, all morphological evaluations were performed by independent assessors who were strictly blinded to the experimental conditions. Further detailed scoring criteria are provided in eMethod 1.

### Bronchoalveolar Lavage Fluid (BALF) analysis

For those pups assigned to biochemical analysis, the left lung was processed with cold saline lavage (15 mL/kg BW each time for three times), and BALF was pooled and the volume measured. BALF was subjected to quantitative measurements of total phospholipids (TPL) and disaturated phosphatidylcholine (DSPC) corrected for BALF volume and BW (details in eMethod 2)^16^. Total proteins (TP) were quantified using BCA kit (Pierce BCA Assay Kit 23225, Thermo Scientific, Rockford, IL).

### RT-PCR analysis of inflammatory mediators, surfactant proteins

The right lung tissue was utilized to quantify mRNA expression via RT-PCR analysis by Roche LightCycler 480 II (Roche, Basel, Switzerland). The mRNA sequence information of target genes was obtained from the NCBI nucleotide databases (www.ncbi.nlm.nih.gov/gene/), and the primers were designed by Sangon Biotech (Shanghai, China). These genes included (1) inflammatory mediators: toll-like receptor (TLR)-4, nuclear transcription factor-kappa B (NF-κB, subunit p50), tumor necrosis factor (TNF)-α, interleukin (IL)-1β, IL-6, IL-8; (2) surfactant-associated genes: surfactant proteins (SP-A, B, C, D), cytidine triphosphate: phosphocholine cytidylyltransferase α (CCTα), secretory phospholipase A2 (sPLA2). The amplification reaction was performed in a 10-µL volume containing 5 µL SYBR^®^Premix Ex Taq™ (Takara Bio Inc, Otsu, Shiga, Japan). The mRNA expression of each gene was normalized to β-actin, using the 2^-ΔΔCT method to calculate relative fold changes. Primer sequences are provided in the supplementary eTable 1.

### Transcriptomic analysis of lung tissues

Right lung samples from animals in the Saline, LPS and LPS-011-675 groups (3–4 samples in each group) were randomly chosen for transcriptomic analysis. RNA-seq data analysis was performed using the NovelBrain Cloud Analysis Platform (NovelBio Bio-Pharm Technology Co., Ltd.). We applied fastp with default parameter filtering the adaptor sequence and removed the low-quality reads to achieve clean data. The clean reads were then aligned to Rabbit genome (OryCun2.0) using the Hisat2^17^. HTseq was used to get raw gene counts. To identify differentially expressed genes (DEGs), the DESeq2 algorithm was strictly applied to the raw count matrices for normalization and differential expression analysis^18^. The Reads Per Kilobase per Million mapped reads (RPKM) method was utilized solely for the determination and visualization of relative gene expression levels^19^. All sequenced samples passed the quality control threshold (RNA Integrity Number > 7.0) and exhibited consistent intra-group clustering (as visually confirmed by heatmap analysis); thus, no outliers were excluded. Differentially expressed genes (DEGs) were identified based on the following criteria: (1) Fold Change > 2 or < 0.5; and (2) *p*-value < 0.05 with a False Discovery Rate (FDR, controlled via the Benjamini-Hochberg procedure) < 0.05^20^. Subsequently, Gene Ontology (GO)^21^ and Kyoto Encyclopedia of Genes and Genomes (KEGG)^22^ analyses were performed using Fisher’s exact test to elucidate the biological processes (BP) and signaling pathways associated with the DEGs. To strictly control for false positives, *p*-values from these enrichment analyses were identically corrected for multiple comparisons, with significantly enriched GO terms and KEGG pathways both defined using a stringent threshold of FDR < 0.05^23^.

### Statistical analysis

Statistical analyses were performed utilizing IBM SPSS Statistics (version 23.0, Armonk, NY, USA) and GraphPad Prism (version 8, La Jolla, CA, USA) for comprehensive data processing and visualization. Survival analysis employed the Kaplan-Meier method with log-rank tests to assess group-level differences. Data normality was rigorously evaluated using the Shapiro-Wilk test, with continuous variables reported as mean *±* standard deviation for parametric data or median with interquartile range for non-parametric distributions. Comparative statistical analyses utilized one-way ANOVA for parametric variables or the Kruskal-Wallis test for non-parametric data, with subsequent pairwise comparisons conducted using Bonferroni post hoc tests or Mann-Whitney U tests, contingent upon variance homogeneity. Categorical variables were presented as counts (percentages) and analyzed by Chi-square test or Fisher’s exact test when appropriate. For the time trend analysis of CIS, a linear mixed model was employed, which included the fixed effects of time, treatment, and their interaction, as well as the random effect of the subjects. The estimated mean differences in CIS between different treatments were also calculated. Statistical significance was set at a *p*-value less than 0.05.

## Results

### CQMUH-011 improved neurological deficits and restored blood gas balance in LPS-induced ALI in neonatal rabbits

**Fig. 1:**
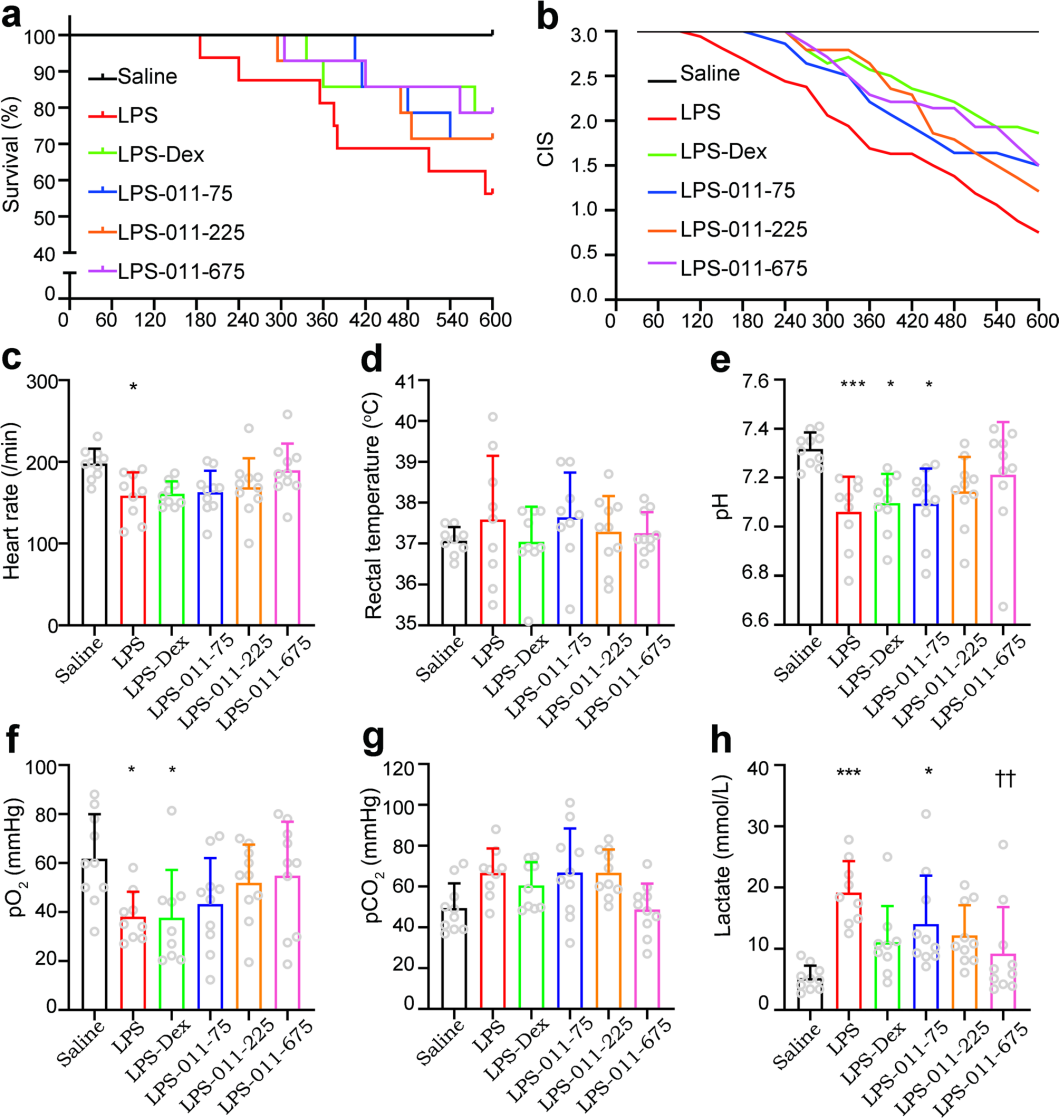
The general conditions of animals among groups. (**a**) Kaplan-Meier survival curve. (**b**) Central injury score (CIS). (**c**) Heart rate. (**d**) Rectal temperature. (**e**) Potential of hydrogen (pH). (**f**) Partial oxygen pressure (pO_2_). **g** Partial carbon dioxide pressure (pCO_2_). (**h**) Lactate concentration. Group definitions and abbreviations: Saline, control; LPS, lipopolysaccharide (50 mg/kg); Dex, dexamethasone (1.5 mg/kg); 011–75, CQMUH-011 at 75 µg/kg; 011–225, CQMUH-011 at 225 µg/kg; 011–675, CQMUH-011 at 675 µg/kg. Values are presented as survival proportion (%) in (**a**), mean in (**b**) (*n* = 14–16 in each group), and mean ± standard deviation in (**c–h**) (*n* = 9–10 in each group). *p < 0.05, **p < 0.01, ***p < 0.001 vs. Saline, ^††^*p* < 0.01 vs. LPS.

A total of 86 pups were enrolled in the study, with no significant differences in BW among all groups (eTable 2). At 10-hour termination, the LPS group exhibited significantly reduced survival rates when compared to the Saline control group (56.3% vs. 100%, *p* = 0.007). In comparison with LPS group, pharmacological interventions (Dex and CQMUH-011 at 75–675 µg/kg) demonstrated a non-significant trend toward improved survival (78.6%, 71.4%, 71.4%, 78.6% vs. 56.3%, *p* > 0.05, respectively) (eTable 2). Kaplan-Meier with Mantel-Cox test confirmed reduced survival duration in LPS-treated animals versus Saline control (*p* = 0.006), though no therapeutic group differed significantly from either Saline or LPS group (Fig. 1a). Notably, both CQMUH-011 and Dex ameliorated sepsis-induced neurological deficits (60–150% improvement over LPS group at 10-hour observation) (Fig. 1b and eTable 2). The high-dose CQMUH-011 group (675 µg/kg) demonstrated superior neuroprotection, in comparison with low-dose CQMUH-011 and Dex groups (25–50% enhancement) (eTable 2). Significant time trend analysis of CIS, compared to Saline control, was seen in LPS, CQMUH-011 and Dex-treated groups (all *p* < 0.001) (Fig. 1b and eTable 3). The estimated mean changes of CIS for all dosages of CQMUH-011 and Dex-treated groups were significantly higher than those in LPS group (*p* < 0.001), with LPS-011-675 group showing the most remarkable improvement (eTable 4). Additionally, animals in LPS group exhibited a decrease in heart rate and a slight increase in rectal temperature, neither of which was observed in the CQMUH-011- or Dex-treated groups (Fig. 1c,d).

Blood gas analysis revealed significant physiological alterations in the LPS group, with notable decreases in blood pH and pO₂, along with marked increases in pCO₂ and lactate levels. Given that these parameters were derived from mixed venous blood, the severe acidemia (low pH) and profoundly elevated lactate serve as robust surrogate indicators of systemic circulatory and perfusion impairment. Consequently, these findings primarily reflect severe systemic tissue hypoperfusion, global microcirculatory hypoxia, and profound metabolic acidosis, rather than isolated pulmonary oxygenation impairment. The CQMUH-011-treated groups exhibited significant improvements in these pathophysiological parameters, with notable increases in blood pO₂ and significant reductions in lactate levels. Notably, the high-dose CQMUH-011 intervention demonstrated better performance on selected physiological parameters compared to the medium- and low-dose groups, and performed comparably to the Dex reference group. This suggested a dose-dependent protective mechanism against LPS-induced ALI (Fig. 1e-h).

### CQMUH-011 mitigated lung inflammatory injury and reduced mRNA expressions of proinflammatory cytokines

**Fig. 2:**
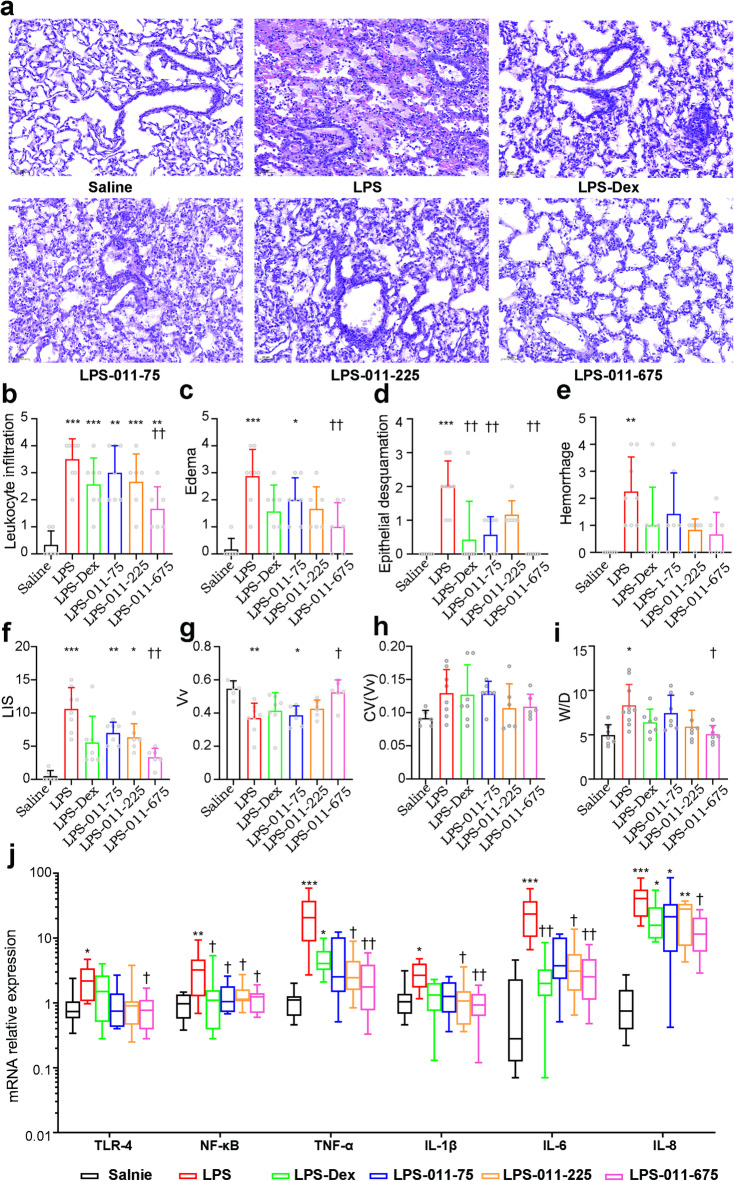
Lung inflammatory injury evaluation and associated mRNA expression among groups. (**a**) Representative photomicrographs of the lungs. HE-stained lung slides under 200-fold amplification. (**b**) Leukcocyte infiltration. (**c**) Edema. (**d**) Epithelial desquamation. (**e**) Hemorrhage. (**f**) Total lung injury scores (LIS). (**g**) Alveolar expansion (Vv). (**h**) Variation of alveolar aeration [CV(Vv)]. (**i**) Wet-to-dry lung weight ratio (W/D). **j** The mRNA expression of proinflammatory mediators from lung tissue. Group definitions see Fig. 1 legends. Abbreviations: TLR, Toll-like receptor; NF-κB, nuclear transcription factor-κB; TNF-α, tumor necrosis factor-α; IL, interleukin. Values are presented as mean ± standard deviation in (**b–i**) (*n* = 6–8 in each group) and median [interquartile range] in (**j**) (*n* = 8–11 in each group). *p < 0.05, **p < 0.01, ***p < 0.001 vs. Saline, ^†^*p* < 0.05, ^††^*p* < 0.01 vs. LPS.

Histopathological evaluation of lung tissue suggested that intraperitoneal LPS administration (50 mg/kg) induced severe ALI, marked by extensive leukocyte infiltration, pulmonary edema, epithelial desquamation, and localized hemorrhagic foci (Fig. 2a). Treatment with CQMUH-011 and Dex demonstrated a substantial mitigation of tissue injury, as evidenced by 40–70% reduction in LIS. The high-dose CQMUH-011 group exhibited the most pronounced therapeutic effect, achieving a maximum average reduction of 70%. Notably, high-dose CQMUH-011 group also presented with better performance in improving alveolar expansion and less pulmonary edema, demonstrated by most improved Vv, CV(Vv) and decreased W/D (Fig. 2b-e).

The RT-PCR results showed that LPS challenge significantly upregulated the mRNA expression of proinflammatory cytokines associated with the TLRs-NF-κB signaling pathway in lung tissue, including TLR-4, NF-κB, IL-1β, IL-6, IL-8, TNF-α (*p* < 0.05). Therapeutic intervention with either Dex or CQMUH-011 effectively suppressed this inflammatory cascade, as evidenced by significant reductions in the abovementioned cytokine transcription levels, with the high-dose CQMUH-011 group showing the most significant attenuation (Fig. 2f).

### **CQMUH-011 enriched phospholipid pool and enhanced the mRNA expressions of associated proteins and enzymes**

**Fig. 3:**
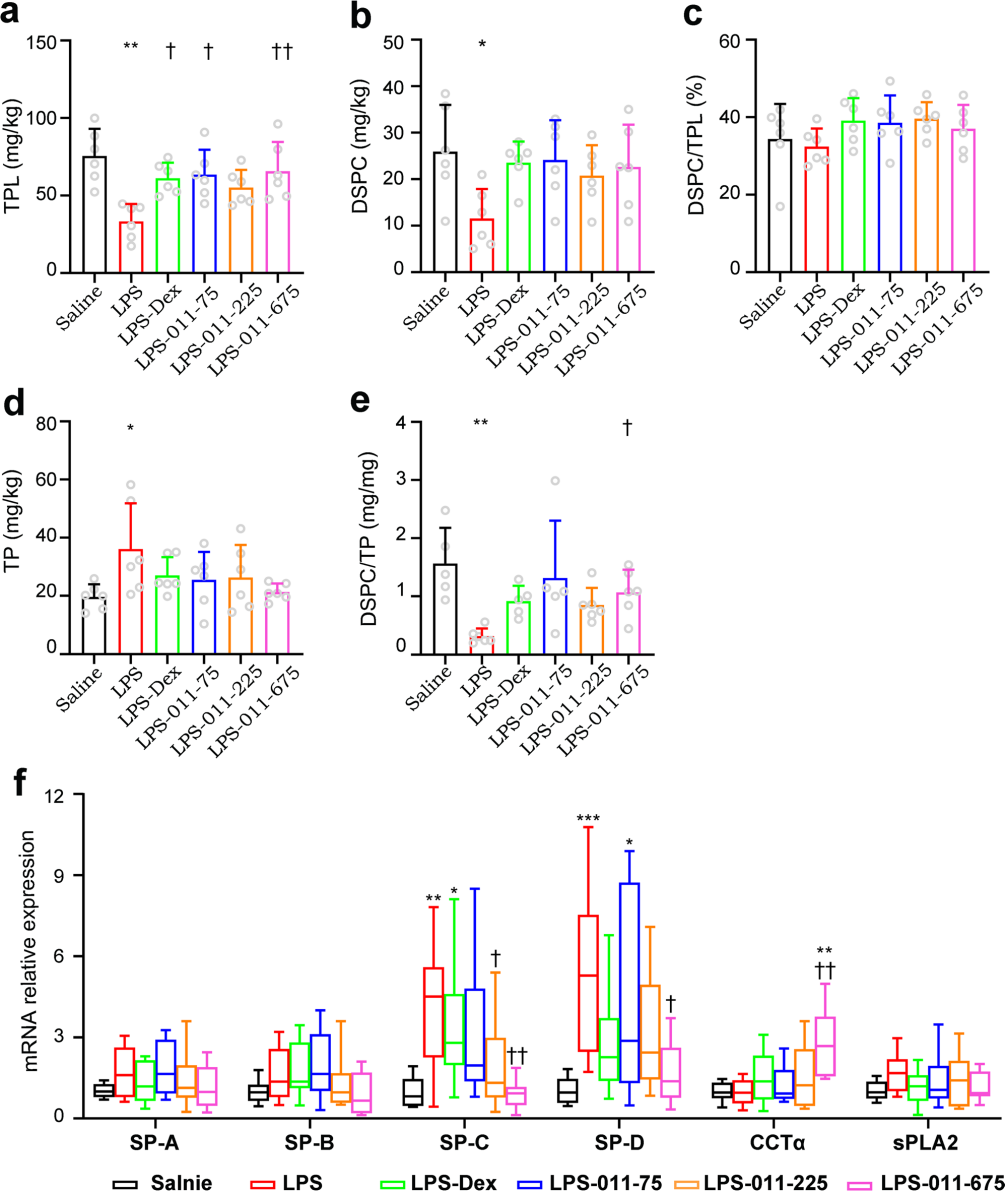
Phospholipid profile and associated mRNA expression. (**a**) Total phospholipids (TPL) in bronchoalveolar lavage fluid (BALF). (**b**) Disaturated phosphatidylcholine (DSPC) in BALF. (**c**) Total proteins (TP) in BALF. (**d**) The ratio of DSPC/TPL. (**e**) The ratio of DSPC/total proteins (TP). (**f**) The mRNA expression of surfactant proteins and associated enzymes from lung tissue. Group definitions see Fig. 1 legends. surfactant proteins, SP; CCTα, cytidine triphosphate: phosphocholine cytidylyltransferase α; sPLA2, secretory phospholipase A2. Values are presented as mean ± standard deviation in (**a–e**) (*n* = 6 in each group) and median [interquartile range] in **f** (*n* = 8–11 in each group). *p < 0.05, **p < 0.01, ***p < 0.001 vs. Saline, ^†^*p* < 0.05, ^††^*p* < 0.01 vs. LPS.

In the LPS group, levels of TPL and DSPC in BALF were significantly reduced while TP significantly increased, compared to Saline control (*p* < 0.05). The effects of LPS on TPL reduction was attenuated by the administration of CQMUH-011 and Dex (*p* < 0.05). Similar trend was observed for DSPC although statistical differences were not reached. There were no significant changes across the groups for DSPC/TPL, whereas DSPC/TP was significantly increased in high-dose CQMUH-011 group (Fig. 3a-e).

LPS intervention induced an upregulation of surfactant protein mRNA expression, with a significant increase in SP-C and SP-D. This upregulation of SP-C and SP-D was suppressed by high-dose CQMUH-011 treatment with a notable decline compared to LPS group (*p* < 0.05). The mRNA expression of surfactant metabolism-associated enzymes suggested that there was no difference in the mRNA expression of CCTα and marginal but non-significant increase in sPLA2 in LPS group, as compared to Saline group. High-dose CQMUH-011 treatment significantly enhanced the mRNA expression of CCTα compared to both Saline and LPS groups (*p* < 0.01), but no significant differences in sPLA2 expression were detected following Dex or CQMUH-011 treatment (Fig. 3f).

### Transcriptome profiling identified gene expression signatures of CQMUH-011 associated with anti-inflammation and cellular protection

**Fig. 4:**
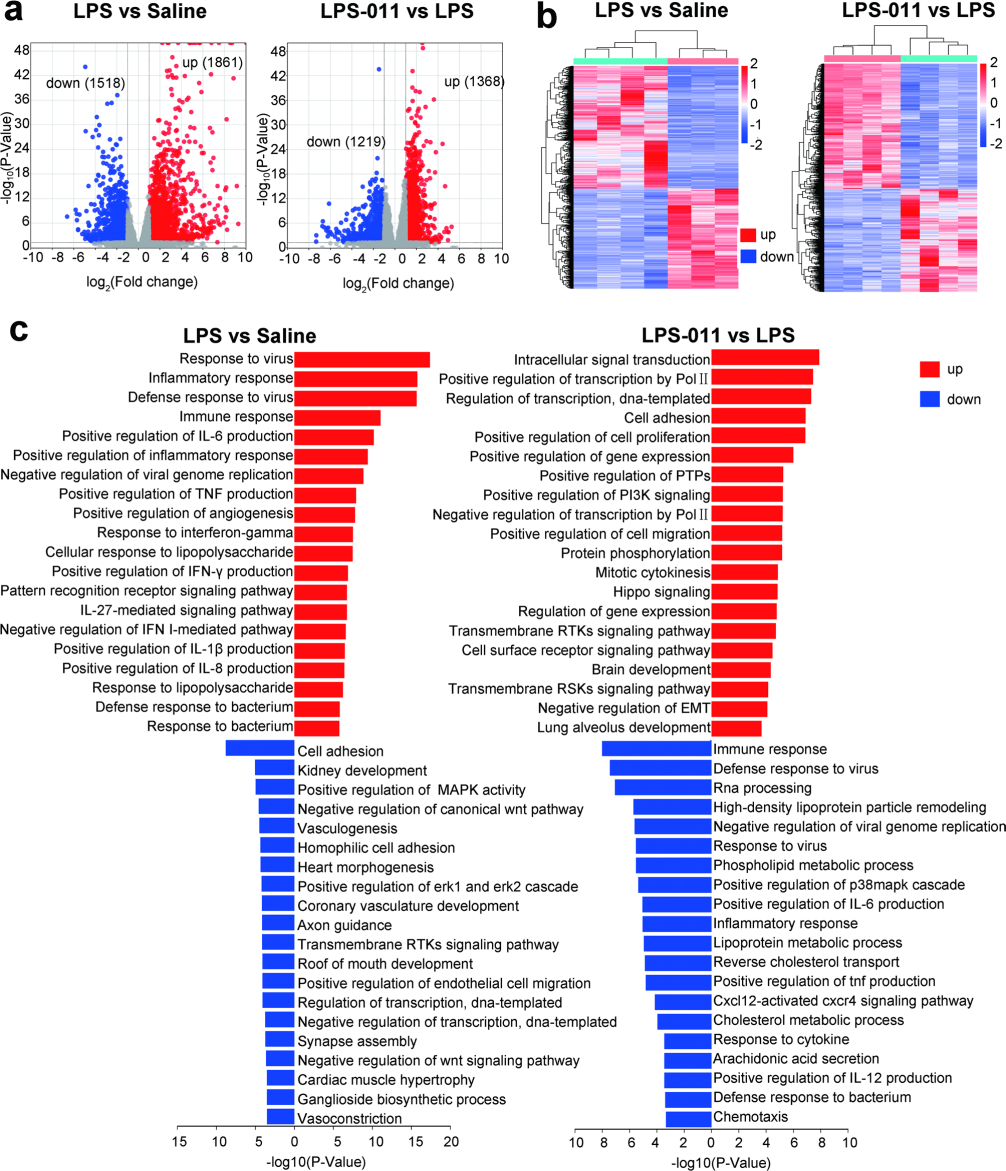
Differentially expressed genes (DEGs) and Go analysis across various treatment groups. (**a**) Volcano plot showing DEGs in LPS vs. Saline (upregulated 1861 genes, downregulated 1518 genes) and LPS-011 vs. LPS (upregulated 1368 genes, downregulated 1219 genes). Significance thresholds were set at an adjusted *p* < 0.05 (FDR < 0.05) and a fold change > 2 or < 0.5. (**b**) Heatmap showing DEGs between groups: LPS vs. Saline and LPS-011 vs. LPS. Red color indicates upregulated DEGs while blue color indicates downregulated DEGs. Each row represents a single DEG, and each column represents an individual sample. (**c**) GO analysis of the top 20 significantly enriched upregulated and downregulated biological process (BP) of DEGs in LPS vs. Saline and LPS-011 vs. LPS groups. Red color indicates upregulated BP while blue color indicates downregulated BP. Group definitions and abbreviations: Saline, control; LPS, lipopolysaccharide (50 mg/kg); 011, CQMUH-011 at 675 µg/kg.

**Fig. 5:**
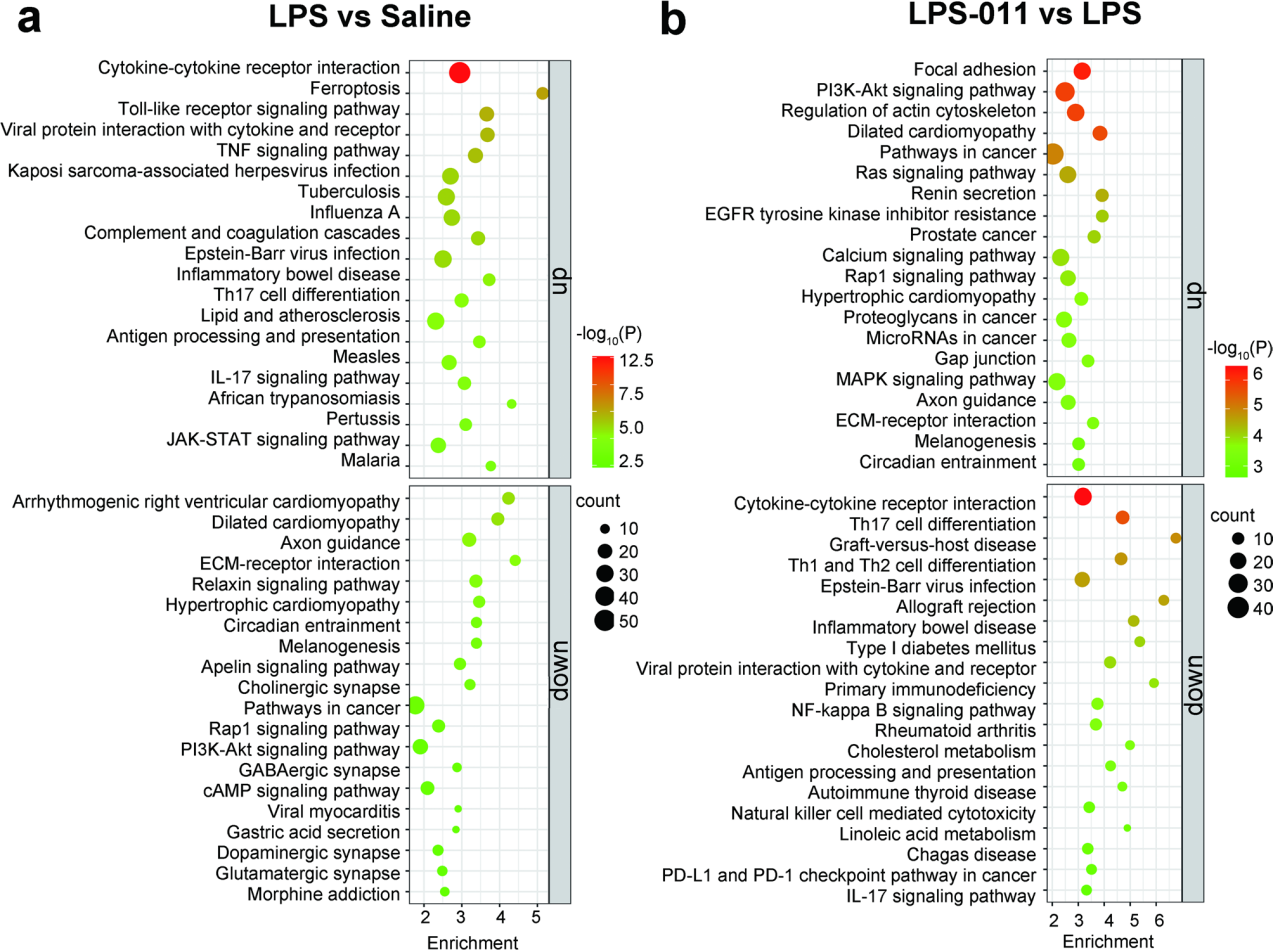
KEGG pathway analysis plot for differentially expressed genes (DEGs) across various treatment groups. (**a**) The top 20 significantly enriched upregulated and downregulated pathways in the LPS vs. Saline group. (**b**) The top 20 significantly enriched upregulated and downregulated pathways in the LPS-011 vs. LPS group. Group definitions and abbreviations see Fig. 4 legends. The bubble size represents the number of differentially expressed genes enriched in the pathway. The color represents the enrichment significance in the pathway, and the larger the value, the more significant the enrichment. The KEGG pathway map is reproduced with permission from Kanehisa Laboratories, Japan (Permission Reference Number: 254526 and 254529).

RNA-seq analysis of neonatal rabbit lungs revealed profound transcriptomic reprogramming following LPS challenge, with 1861 upregulated and 1518 downregulated DEGs versus Saline group. Treatment with high-dose CQMUH-011 (675 µg/kg) demonstrated 1219 downregulated and 1368 upregulated genes versus LPS group (Fig. 4a-b). GO analysis identified the 20 most significant BP across different intervention groups. The LPS group exhibited upregulation of genes associated with lung inflammation (including IL-6 and TNF-α), immune response, and pro-angiogenic processes. Following intervention with CQMUH-011, a notable downregulation was observed in inflammation-related genes, p38 mitogen-activated protein kinase (p38MAPK) regulation, and immune response-associated transcripts (e.g., IL-6, IL-12, chemotaxis). Additionally, multiple metabolic processes were also downregulated, including phospholipid, lipoprotein, and cholesterol metabolism. It is worth noting that CQMUH-011 effectively mitigated the downregulation of cell adhesion molecules induced by LPS (Fig. 4c). KEGG Pathway analysis indicated that CQMUH-011 potentially upregulated pathways associated with anti-inflammatory responses and cell survival, specifically the phosphatidylinositol 3-kinase/protein kinase B (PI3K-AKT) pathway, as well as pathways involved in cytoskeletal regulation, including actin cytoskeleton organization, focal adhesion, gap junction, and Rap1 signaling, thereby enhancing cellular stability and repair mechanisms. Simultaneously, transcriptomic signatures suggested that CQMUH-011 downregulated pro-inflammatory pathways, such as cytokine-cytokine receptor interactions, T-helper 17 (TH17) cell differentiation, and the IL-17, TNF-α, and NF-κB signaling pathways, effectively mitigating excessive immune activation (Fig. 5a-b).

## Discussion

This study demonstrated that intraperitoneal administration of LPS (50 mg/kg) in neonatal rabbits induced a severe, hyper-acute systemic endotoxin shock paradigm that drives secondary indirect ALI. Designed to serve as a surrogate for the early pathological cascade of sepsis-associated ALI, in the absence of mechanical respiratory support and fluid/vasoactive resuscitation, this model successfully recapitulated key clinical features, including increased mortality, compromised neuromotor function, lactic acidosis (indicative of tissue hypoperfusion), respiratory failure, and severe lung injury with typical pathological and molecular biological changes. Early intervention with CQMUH-011 resulted in improvements in neuromotor dysfunction, enhanced oxygenation, reduced metabolic acidosis, and significant amelioration of lung pathological damage. It also suppressed the mRNA expression of inflammatory cytokines, and restored the dysfunction and dysregulation of phospholipid synthesis. Transcriptome sequencing further confirmed that CQMUH-011 intervention significantly downregulated the expression of multiple inflammatory signaling pathways induced by LPS in the lungs, including cytokine-cytokine receptor interaction, TH17 cell differentiation, and IL-17/NF-κB/TNF-α pathways, while enhancing pathways associated with anti-inflammation, cellular stability and repair. Moreover, the study identified a potential dose-dependent anti-inflammatory response to CQMUH-011, with comparable therapeutic efficacy in lung protection compared to Dex intervention. These findings suggest that CQMUH-011 represents a promising therapeutic agent for inflammation-related diseases, particularly through its dual role in anti-inflammation and cellular protection, offering a complementary mechanism to traditional glucocorticoid therapies like Dex. It should be noted that the 10-hour observation window utilized in this study was specifically selected to capture the hyper-acute phase of the LPS-induced cytokine storm and early lung injury. Extending the observation in this severe, high-dose model typically leads to confounding terminal multi-organ failure, complicating the precise assessment of lung-specific damage and surfactant biology.

Neonatal sepsis-associated ARDS is one of the leading causes of morbidity and mortality in preterm and term infants^5–7^. A dysregulated host inflammatory response, disruption of the alveolar-capillary barrier, and impairment of surfactant homeostasis contribute to the pathogenesis^24–27^. In adult ARDS, various anti-inflammatory strategies, including corticosteroids, vitamins, satins, N-Acetylcysteine, surfactant, and nitric oxide, have been explored and have shown some benefits^28,29^. Although bench and animal researches have demonstrated potential benefits in neonatal sepsis-induced ARDS, the use of anti-inflammatory therapy in the neonates has been limited due to the lack of adequate data in its safety and efficacy in this population^15,16,30,31^. Of particular concern, increased susceptibility to secondary infection, restriction on growth and neurodevelopment are major concerns in the use of corticosteroids^8,32^. Therefore, there is an urgent need to explore novel therapeutic strategies that are safe and can effectively mitigate the inflammatory cascades underlying neonatal sepsis-associated ARDS.

CQMUH-011, a novel small-molecule anti-inflammatory agent, has demonstrated comparable efficacy to dexamethasone in inhibiting inflammatory responses in various experimental settings. This includes in vitro studies using LPS-challenged macrophages, as well as in vivo models of inflammatory injury in the brain, liver, and arthritis/osteoarthritis models^9–12^. Toxicity experiments have been used to verify its safety. Acute toxicity testing of CQMUH-011 (40 times of therapeutic dose, intraperitoneal injection) induced only transient somnolence in the first 2 h in mice, with full behavioral recovery and 100% survival at 14 days. Histopathological evaluation revealed no abnormalities in pathological dissection of various organs^9^. The present study demonstrates that CQMUH-011 possessed potent anti-inflammatory and lung-protective properties in a neonatal rabbit model of LPS-induced ALI. Specifically, CQMUH-011 significantly improved blood gas parameters, and lung histopathology in a dose-dependent manner, performing comparably to dexamethasone, or better on selected endpoints, though the survival benefit did not reach statistical significance. These findings are consistent with previous reports showing the anti-inflammatory and organ-protective effects of CQMUH-011^10–12^.

Importantly, our transcriptomic profiling provides only a descriptive landscape of mRNA-level changes. Because we lack “hard” protein-level and functional validation, the exact molecular mechanisms remain unconfirmed. The following networks underlying CQMUH-011’s therapeutic efficacy are strictly hypothesized transcriptomic signatures rather than proven pathways: (1) Hypothesized TLR4/NF-κB axis suppression: CQMUH-011 attenuated LPS-induced TLR-4 upregulation, inhibiting NF-κB activation and pro-inflammatory cytokine release (e.g., TNF-α, IL-1β, IL-6)^4^, consistent with its anti-inflammatory effects in macrophages and microglia^9,12^. (2) Potential cytokine network modulation: Downregulation of cytokine-cytokine receptor interaction pathways disrupted inflammatory amplification loops, stabilizing alveolar-capillary barriers and surfactant metabolism^4,26,27^. (3) Hypothesized TH17/IL-17 pathway inhibition: Suppression of genes involved in the TH17/IL-17 signaling cascade mitigated neutrophilic infiltration, epithelial/endothelial cell dysfunction and tissue damage^33,34^, mirroring clinical benefits of IL-17 blockade in ARDS trials^31^. (4) Hypothesized PI3K-AKT-mediated cytoprotection: Upregulation of PI3K-AKT pathway, has been shown to exert protective effects in the context of experimental ALI by promoting cell survival, suppressing apoptosis, and enhancing the resolution of inflammation^24,35^. The PI3K-AKT pathway also involved in the restoration of neurological function^36^, consistent with our current findings of the improved neurological status in CQMUH-011 groups. (5) Potential cytoskeletal-architectural restoration: Coordinated activation of focal adhesion, gap junction, and Rap1 signaling reinforced endothelial integrity and promoted alveolar repair via basement membrane remodeling^37,38^. (6) Potential metabolic reprogramming in inflammatory regulation: Emerging evidence underscores critical interactions between immunometabolic networks and inflammatory pathogenesis^39^. CQMUH-011 significantly modulated key lipid regulatory axes, demonstrating coordinated downregulation of phospholipid, lipoprotein and cholesterol homeostasis, which appears to disrupt pro-inflammatory lipid signaling cascades while restoring immunometabolic balance. The ability of CQMUH-011 to simultaneously dampen pro-inflammatory cascades and enhance anti-inflammation, cytoskeleton regulation and tissue repair signaling may contribute to its potent lung protective effects in neonatal ALI.

Our findings suggest that CQMUH-011 not only mitigates inflammation but also preserves pulmonary surfactant homeostasis, a critical determinant of neonatal lung function and recovery. In neonatal ALI, surfactant dysfunction, as characterized by impaired synthesis and altered composition, contributes to alveolar collapse and respiratory failure^4,26^. Notably, CQMUH-011 treatment significantly elevated TPL and DSPC in BALF compared to the LPS group, indicating restored surfactant homeostasis. At the transcriptional level, LPS challenge induced a dramatic upregulation of SP-C and SP-D mRNA. Consistent with previous literature, this likely represents a rapid compensatory acute-phase response by alveolar type II epithelial cells attempting to counteract severe inflammatory stress and acute surfactant depletion^27^. High-dose CQMUH-011 (675 µg/kg) effectively blunted this compensatory mRNA spike. Rather than directly inhibiting a beneficial pathway, this suppression likely reflects a profound reduction in the primary inflammatory insult. By mitigating the early cytokine storm, CQMUH-011 relieves the extreme cellular stress on the pulmonary epithelium, thereby reducing the physiological requirement for compensatory SP overexpression. Concurrently, while the mRNA expression of CCTα, a surfactant metabolism-related enzyme, showed no differences between LPS and saline groups, high-dose CQMUH-011 uniquely upregulated CCTα expression, suggesting enhanced surfactant synthesis. Interestingly, our transcriptomic GO analysis indicated a broad downregulation of generalized phospholipid and lipoprotein metabolism following CQMUH-011 treatment. While seemingly contradictory to the biochemical recovery of TPL, this transcriptomic shift likely reflects critical immunometabolic reprogramming during acute inflammation^40^. Specifically, CQMUH-011 appears to shut down highly active, energy-consuming pro-inflammatory lipid signaling cascades (e.g., arachidonic acid and inflammatory lipid mediator pathways). By halting this pathological metabolic overdrive and simultaneously upregulating CCTα, the lung is able to preserve its structural surfactant pool. The robust restoration of surfactant homeostasis observed with high-dose CQMUH-011 underscores its therapeutic potential in stabilizing alveoli and improving respiratory outcomes in neonatal ALI.

The present study has several limitations. Firstly, our neonatal *in vivo* model remains insufficiently characterized at the systemic level. Because our experimental design aimed to observe the natural, unsupported early course of the disease, it lacked mechanical respiratory support and fluid/vasoactive resuscitation. This absence of standard life support heavily drove the observed hyper-acute endotoxin shock phenotype and early mortality. While our mixed venous blood gas data (particularly severe acidemia and profoundly elevated lactate) serve as robust surrogate indicators for systemic tissue hypoperfusion, we acknowledge the absence of broader sepsis-related readouts. Specifically, the lack of complete peripheral blood cell counts, protein-level systemic inflammatory mediators, direct invasive hemodynamic measurements, and comprehensive multi-organ injury markers limits our ability to fully profile this systemic shock syndrome. Therefore, future investigations utilizing this hyper-acute endotoxin shock paradigm must incorporate these comprehensive systemic readouts alongside standard life support to better simulate clinical sepsis-associated indirect ALI. Furthermore, “litter” was not explicitly modeled as a random effect, and the study was not statistically powered to evaluate sex-specific differences. Secondly, the short 10-hour observation window limits the assessment of long-term outcomes. While CQMUH-011 treatment yielded a notable absolute numerical improvement of 25–40% in early survival, it did not reach statistical significance. However, given the profound alleviation of lung injury observed at the histological and molecular levels within this short timeframe, we hypothesize that extended observation periods (e.g., 24–72 h) might reveal significant survival benefits. Consequently, the capacity of CQMUH-011 for sustained alveolar repair, chronic safety, and long-term neurodevelopment remains unexplored. Therefore, despite performing comparably to or better than dexamethasone on selected acute endpoints, definitive clinical superiority cannot be inferred without long-term safety data. Thirdly, certain methodological constraints restrict mechanistic interpretations. The reliance on mixed venous blood precludes the precise calculation of arterial oxygenation indices (e.g., PaO_2_/FiO_2_). Although our transcriptomic GO and KEGG enrichment analyses were stringently controlled using multiple-testing correction (FDR < 0.05), the relatively small sample size for RNA-seq (*n* = 3–4 per group) combined with the exclusive reliance on mRNA-level data means the proposed signaling networks (e.g., TLR4/NF-κB, IL-17/Th17, PI3K-AKT) cannot be definitively confirmed. Due to the limited tissue yield from neonatal rabbits and a lack of validated cross-reactive antibodies, this study lacks functional and protein-level validations (e.g., p-p65/p-AKT, cytokine ELISAs, Evans blue permeability, and tight junction proteins). Consequently, the proposed signaling pathways must be interpreted strictly as exploratory, hypothesis-generating networks requiring future experimental confirmation. Finally, the translational framing of this study is strictly limited to early proof-of-concept. At this stage, there is still no precise pharmacokinetic (PK) characterization (e.g., ADME profiling, half-life, and tissue distribution) of CQMUH-011 in the neonatal system. Furthermore, we have not conducted any neonatal-specific safety assessments. Given the extreme vulnerability of the developing neonatal brain and organs, future studies must rigorously evaluate potential neurodevelopmental toxicity, off-target effects, and long-term safety before CQMUH-011 can be genuinely presented as a viable therapeutic candidate for neonatal or pediatric clinical translation.

In conclusion, the present study demonstrates that the novel compound CQMUH-011 exerts potent anti-inflammatory and lung-protective effects in a neonatal rabbit model of LPS-induced ALI. The therapeutic efficacy of CQMUH-011 appears to be mediated, at least in part, through the modulation of key inflammatory signaling pathways, preservation of surfactant homeostasis, and enhancement of lung repair programs. These findings highlight the preliminary protective effects of CQMUH-011 against endotoxin-induced secondary lung injury in neonates, warranting further exploration in more clinically relevant sepsis models. Importantly, indispensable pharmacokinetic profiling and stringent neonatal-specific safety evaluations are strictly required before framing this compound as a viable therapeutic candidate.

## Supplementary Information

Below is the link to the electronic supplementary material.

Supplementary Material 1

## Data Availability

The dataset(s) supporting the findings of this study have been deposited in the Gene Expression Omnibus (GEO) repository under accession number GSE312949. These data can be accessed via: https://www.ncbi.nlm.nih.gov/geo/query/acc.cgi?acc=GSE312949 (Reviewers Token: wvahsccezdkdhch). Data will be released upon publication.
