# Supplementary material for: CQMUH-011 mitigated LPS-induced acute lung injury in neonatal rabbits
Source: Sci Rep. 2026 Apr 19;16:18129. doi: 10.1038/s41598-026-49167-x (PMC13253820; doi:10.1038/s41598-026-49167-x)
Supplement: Supplementary file 1 — Supplementary Material 1 [file 41598_2026_49167_MOESM1_ESM.pdf]

Supplementary information

eMethod 1. Measurements of lung histological parameters.

eMethod 2. Measurements of phospholipids in bronchoalveolar lavage fluid.

eTable 1. Primer sequences (5'-3') of target genes (F: forward, R: reverse).

eTable 2. The general conditions of animals at 10 h of observation.

eTable 3. The trend analysis of central injury score (CIS) by linear mixed model.

eTable 4. Estimates of the mean difference using multiple comparisons of the means of central injury score (CIS) between groups by linear mixed model.

eMethod 1. Measurements of lung histological parameters.

The accessory lobe of the right lung was removed and its wet weight was determined in an automatic electric balance (AP250D; Ohaus, Florham, NJ). The piece of the lung tissue was then put in an oven at 80ºC for 72 hours and weighed again to obtain its dry weight for calculation of the wet-to-dry weight ratio (W/D).

In all groups, the left lung was perfusion fixed initially by 60 ml of cold saline and 4% paraformaldehyde via the pulmonary artery catheter for 10 min. Then, the left lung was dissected and further fixed with 4% paraformaldehyde for 24 h, and embedded in paraffin, followed by section and stain with hematoxylin and eosin (HE). The lung sections were examined under light microscope (Leica Microsystems, Wetzlar, Germany). Lung injury score (LIS) for lung histopathologic changes comprised four items (leukocyte infiltration, edema, hemorrhage, bronchiole epithelial desquamation) and total score. A 4-score scale was used to represent the severity of lung injury: 0 for no or very minor (<1% area), 1 for modest and limited (1-25%), 2 for intermediate (26-50%), 3 for widespread or prominent (51-75%), 4 for widespread and most prominent (>75%). A point-counting method was applied to quantify lung expansion, which was expressed as volume density (Vv) of aerated alveolar spaces with total parenchyma as the reference volume. Fifty fields of the lung tissue from each animal lung were examined at ×200 magnification, and field-to-field variability was determined by calculating the coefficient of variation of Vv (CV[Vv]). All the morphological evaluation was performed blindly, i.e. without knowledge of the experimental condition of individual animals.

eMethod 2. Measurements of phospholipids in bronchoalveolar lavage fluid.

Sterile saline at a volume equivalent to 15 ml/kg birthweight (BW) was used for left lung bronchoalveolar lavage (BAL) by three repeats. The returned BAL fluid (BALF) was pooled and immediately centrifuged at 2,000 rpm for 15 min at 4°C to remove cell debris and its supernatant was frozen at -20°C for further use.

TPL were extracted by chloroform-methanol (vol/vol 2:1) from BALF (supernatant), separately. DSPC was recovered by the neutral alumina column chromatography after exposing the isolated TPL to osmium tetroxide. TPL and DSPC were quantified with the molybdenum acid method for inorganic phosphorus determination (Pi, μg) through colorimetry. The calculation formula included:

VBALF (ml) = VL/0.45

(VL: volume of left lung BALF; 0.45: the proportion of left lung of total lungs)

TPL or DSPC in BALF with BW correction (mg/kg) = Pi *25*VBALF*10/BW

(25: the conversion factor of Pi translating into organic phosphorus)

eTable 1. Primer sequences (5’-3’) of target genes (F: forward, R: reverse)

Target gene	F/R primer	Primer sequences
β-actin	F	ATTGGCATGGCTTTATTCGTG
	R	GTCACCTTCACCGTTCCAGTTT
TLR-4	F	GTATCGCCTTCTCAGCAGGAACAC
	R	TGAGCCGTCTCCAGAAGATATGCC
NF-κB	F	GATGTGAAGATGCTCCTGGCTGTC
	R	CGGTGGATGATTGCTAGGTGTAAGAC
TNF-α	F	GGCATGAAGCTCACGGACAACC
	R	GCCTTGACCGCTGAAGAGAACC
IL-1β	F	CAGGCTCCAGGATGCACAACAG
	R	CACGCAGGACAGGTACAGATTCTTC
IL-6	F	GAAGAAGCCACCCTCAAGCC
	R	CCATGAAATTCCGCAAGCAA
IL-8	F	TTGGTCAGGCCATGAGTTCC
	R	TGTGCCCTCACAACATCCCT
SP-A	F	ATGACTCAAGCGTAATAAATCGTG
	R	CTCTGGACAAAGCGAGCACA
SP-B	F	GCTTTACCGCTACTTGCTCACA
	R	CTTGGACCATCTTCTGTTGCTTT
SP-C	F	TGGTCCTCGTGGTCGTGGTG
	R	GCTGGTAGTCGCAGGTGACAATG
SP-D	F	CCTGAGCATGACTGACACCAAGAC
	R	CACAAGGCGTTCCTCTCCACAAG
CCT	F	TGACGAGCTGACGCACAACTTC
	R	CCTCACCACTTCATCCACATAGCG
sPLA2	F	CACCGCCACGACTGCTGTTAC
	R	ACCTGCTGACCGACACACTCC

Abbreviations: TLR, toll-like receptor; NF-κB, nuclear transcription factor-κB; TNF-α, tumor necrosis factor-α; IL, interleukin; SP, surfactant proteins; CCT, cytidine triphosphorylate: phosphocholine cytidylyl transferase; sPLA2, secretory phospholipase A2.

eTable 2. The general conditions of animals at 10 h of observation.

	N	Body weight	Survival, n (%)	CIS at 5 h	CIS at 10 h
Saline	14	101$\pm$7.4	14 (100)	3$\pm$0	3$\pm$0
LPS	16	95.8$\pm$7.5	9 (56.3)	2.06$\pm$1.06*	0.75$\pm$0.77***
LPS-Dex	14	94.7$\pm$9.1	11 (78.6)	2.71$\pm$0.61	1.50$\pm$1.22**†
LPS-011-75	14	96.6$\pm$7.1	10 (71.4)	2.79$\pm$0.43	1.21$\pm$1.05***
LPS-011-225	14	94.4$\pm$5.4	11 (78.6)	2.57$\pm$0.85	1.50$\pm$1.29**†
LPS-011-675	14	95.3$\pm$4.9	11 (78.6)	2.64$\pm$0.84	1.86$\pm$1.17††

Group definitions and abbreviations: Saline, control; LPS, lipopolysaccharide (50 mg/kg); Dex, dexamethasone (1.5 mg/kg); 011-75, CQMUH-011 at 75 μg/kg; 011-225, CQMUH-011 at 225 μg/kg; 011-675, CQMUH-011 at 675 μg/kg; CIS, central injury score. Values are presented as number (proportion) or mean $\pm$ SD. **p* < 0.05, ***p* < 0.01, ****p* < 0.001 *vs.* Saline, †*p* < 0.05, ††*p* < 0.01 *vs.* LPS.

eTable 3. The trend analysis of central injury score (CIS) by linear mixed model.

Fixed effects	CIS estimates (95% confidence interval)	*p* value
Intercept	3.000 (2.822, 3.178)	<0.001
Group		
LPS	0.367 (0.124, 0.610)	0.003
LPS-Dex	0.367 (0.115, 0.618)	0.004
LPS-011-75	0.510 (0.259, 0.761)	<0.001
LPS-011-225	0.393 (0.142, 0.644)	0.002
LPS-011-675	0.309 (0.058, 0.560)	0.016
Saline	0	
Time (hour)	0.000 (-0.030, 0.030)	1.000
Group*Time (hour)		
LPS*Time	-0.256 (-0.296, -0.215)	<0.001
LPS-Dex*Time	-0.164 (-0.206, -0.121)	<0.001
LPS-011-75*Time	-0.202 (-0.244, -0.160)	<0.001
LPS-011-225*Time	-0.194 (-0.236, -0.152)	<0.001
LPS-011-675*Time	-0.136 (-0.178, -0.094)	<0.001
Saline*Time	0	

Group definitions and abbreviations see eTable 2 legends.

eTable 4. Estimates of the mean difference using multiple comparisons of the means of central injury score (CIS) between groups by linear mixed model.

Group comparison	Estimated change of CIS (95% confidence interval)	*p* value
LPS *vs.* Saline	-0.975 (-1.150 -0.800)	<0.001
LPS-Dex *vs.* Saline	-0.493 (-0.674, -0.312)	<0.001
LPS-011-75 *vs.* Saline	-0.550 (-0.731, -0.369)	<0.001
LPS-011-225 *vs.* Saline	-0.625 (-0.806, -0.444)	<0.001
LPS-011-675 *vs.* Saline	-0.407 (-0.588, -0.226)	<0.001
LPS-Dex *vs.* LPS	0.482 (0.307, 0.658)	<0.001
LPS-011-75 *vs.* LPS	0.425 (0.250, 0.600)	<0.001
LPS-011-225 *vs.* LPS	0.350 (0.175, 0.525)	<0.001
LPS-011-675 *vs.* LPS	0.568 (0.392, 0.743)	<0.001
LPS-011-75 *vs.* LPS-Dex	-0.057 (-0.238, 0.124)	1.000
LPS-011-225 *vs.* LPS-Dex	-0.132 (-0.313, 0.049)	0.482
LPS-011-675 *vs.* LPS-Dex	0.086 (-0.095, 0.267)	1.000
LPS-011-225 *vs.* LPS-011-75	-0.075 (-0.256, 0.106)	1.000
LPS-011-675 *vs.* LPS-011-75	0.143 (-0.038, 0.324)	0.308
LPS-011-675 *vs.* LPS-011-225	0.218 (0.037, 0.399)	0.006

Group definitions and abbreviations see eTable 2 legends.
